# Ending the HIV/AIDS Pandemic^1^

**DOI:** 10.3201/eid2403.171797

**Published:** 2018-03

**Authors:** Robert Walter Eisinger, Anthony S. Fauci

**Affiliations:** National Institutes of Health, Bethesda, Maryland, USA

**Keywords:** pandemics, acquired immunodeficiency syndrome, AIDS, global health, communicable diseases, HIV infections, life, HIV/AIDS, viruses

## Abstract

The goal of ending the HIV/AIDS pandemic is theoretically achievable and would require addressing this global health catastrophe on individual and global levels by providing optimal prevention strategies and treatment regimens for individual persons living with or at risk for HIV, as well as ending the pandemic as an epidemiologic and global health phenomenon. However, from a practical standpoint, the pathway to ending the HIV/AIDS pandemic will be difficult and will require aggressive implementation of the biomedical research advances that have been made in the areas of treatment and prevention; development of additional tools, such as a moderately effective HIV vaccine; and attention to critical behavioral and social determinants. An end to the HIV/AIDS pandemic can be achieved only with provision of sustained and additional resources at the local, regional, national, and global levels.

Ending the HIV/AIDS pandemic continues to represent a realistic goal that demands that we address numerous considerations and obstacles at the local, regional, national, and global levels. One can view the attainment of this goal from at least 2 separate, but interrelated, vantage points: 1) optimizing treatment and prevention for individual persons living with or at risk for HIV, and 2) ending the HIV/AIDS pandemic as an epidemiologic and global health phenomenon. 

In this article, we describe how the pathway to ending the HIV/AIDS pandemic will involve implementation of each of these elements. The phrase “HIV/AIDS pandemic” indicates that there is a global HIV epidemic, which may be generalized in some countries, such as South Africa, and localized in other countries, such as the United States. The phrase “end of HIV/AIDS” does not refer to the eradication of HIV; rather, it refers to the end of HIV/AIDS as an epidemiologically defined term that meets other criteria. Specifically, the number of new HIV infections would be fewer than the number of AIDS-related deaths; HIV infection and AIDS incidence would decrease to <1 AIDS case/1,000 population; 73% of persons living with HIV and AIDS would be receiving treatment; 86% would experience virus suppression; and mother-to-child transmission would be eliminated to <5% (*1*). Although our comments focus primarily on biomedical research advances and the effects of their implementation, an end to the HIV/AIDS pandemic will require their implementation within the broader socioeconomic, cultural, demographic, and political context at the local, regional, and national levels.

Science and the implementation of its discoveries has and will continue to benefit persons living with HIV and at risk for acquisition of HIV and will drive the process of ending HIV/AIDS as a global pandemic. Since the first reports in 1981 of the unusual cluster of Kaposi sarcoma and *Pneumocystis* pneumonia among young, previously healthy homosexual men in New York, NY, and California, USA (*2**,**3*), substantial scientific advances have been made in the fight against HIV/AIDS. The co-discovery of HIV as the causative agent of AIDS by the Institut Pasteur team led by Montagnier and Barré-Sinoussi (*4*) and the US National Institutes of Health team lead by Gallo (*5*) was merely the beginning of the process. Their seminal findings launched an intensive global multidisciplinary research effort that has led to, and continues to result in, the development and implementation of innovative treatment regimens, prevention interventions, and behavior strategies to extend the healthy lives of those who are already infected, to prevent infection of those at high risk, and to halt and reverse the trajectory of the pandemic. 

## Optimizing Treatment and Prevention for Individual Persons Living with or at Risk for HIV

### Treatment 

Since the early 1980s, major advances have been made with treatment for persons living in HIV. During the subsequent years since the recognition of this new and devastating disease, numerous landmark studies have resulted in availability of >30 US Food and Drug Administration–approved antiretroviral drugs for the treatment of HIV infection, along with an extensive group of strategies for the prevention and treatment of HIV-associated co-infections and comorbidities. 

Over the past 4 decades, implementation of treatment modalities has faced several challenges. The challenges have been associated with drug toxicities, inconsistent adherence to complex treatment regimens, drug resistance, decisions when to initiate treatment, pill fatigue, and limited access to treatment by special populations (*6**,**7*). For persons living with HIV who have access to these drugs, these regimens extended their life expectancy. In 1981 and 1982, when the first AIDS patients were seen in the United States, the median survival time for a person with AIDS was 1–2 years (*8**,**9*). In contrast, for a person of 20-some years of age with HIV infection being treated with combination antiretroviral therapy (ART) today, the projected life expectancy is ≈53 years (*10*). This major accomplishment resulted from public–private partnerships among academia, industry, the US government, international collaborators, and the affected community. According to recent reports from the Joint United Nations Programme on HIV/AIDS (UNAIDS) and largely as a result of the efforts of the US President’s Emergency Plan for AIDS Relief and the Global Fund to Fight AIDS, Tuberculosis and Malaria, 19.5 million persons are now receiving lifesaving ART (representing 53% of all persons living with HIV globally), and AIDS-related deaths have been halved since 2005 (*11*). Increasing the number of persons living with HIV who receive ART will require continued optimization of treatment regimens and implementation of strategies to ensure prompt diagnosis, access to ART, adherence to drug regimens, and retention in care.

### Prevention 

Substantial progress has also been made in the development of strategies to prevent HIV transmission/acquisition, beyond the interventions that were available early in the AIDS epidemic (e.g., use of condoms, clean syringes, behavioral interventions, and blood supply screening). The HIV Prevention Trials Network 052 clinical trial, which involved 1,763 HIV-serodiscordant couples in 9 countries, clearly validated the concept of treatment as prevention as an effective HIV prevention strategy. The initial findings from this landmark clinical study showed a 96% reduction in HIV transmission (from 350–550 to <250 CD4+ T cells/μL) when the HIV-infected partner began ART early (*12*). These results were sustained in a follow-up study 4 years later (*13*). The results from the recent PARTNER study further demonstrated the benefits of suppressive ART in preventing HIV transmission during sexual activity (*14*). From this study, Rodger et al. reported that, after ≈58,000 condomless sex acts among HIV-serodiscordant couples, no linked HIV transmissions occurred while the HIV-infected partner was receiving suppressive ART. In addition, the findings from the Opposites Attract study (358 HIV-serodiscordant couples in Australia, Thailand, and Brazil) recently indicated no HIV transmissions among 591 couple-years of follow-up when the HIV-infected partner had an undetectable viral load (results based on 16,889 acts of condomless anal intercourse) (*15*). Together, these studies demonstrate that when ART effectively suppresses a person’s viral load to undetectable levels, the risk for sexual transmission of HIV to an uninfected sexual partner is essentially zero. These clinical trials provided the crucial scientific evidence for the recent consensus statement that undetectable = untransmittable is achievable if the viral load of a person living with HIV and receiving ART is undetectable (*16*). The US Centers for Disease Control and Prevention recently stated, “When ART results in viral suppression, defined as less than 200 copies/mL or undetectable levels, it prevents sexual HIV transmission” (*17*). Accomplishing the undetectable = untransmittable strategy worldwide will require that everyone who is HIV-infected is receiving ART and that their viral load is suppressed to an undetectable level.

Another valuable HIV-prevention strategy is preexposure prophylaxis (PrEP). Results from several key clinical trials, including iPrEX (*18*), TDF2 (*19*), Partners PrEP (*20*), and the Bangkok Tenofovir Study (*21*), have demonstrated that emtricitabine and tenofovir disoproxil fumarate (Truvada; Gilead Sciences, Inc., Foster City, CA, USA) taken as a single pill daily is ≈95% effective for preventing HIV acquisition. Marcus et al. recently reported that no HIV infections were acquired during 5,104 person-years of PrEP use (*22*). Although PrEP is highly effective as a prevention intervention, it is currently underutilized in the United States and worldwide; thus, public health officials worldwide need to continue to develop, optimize, and implement HIV prevention strategies for persons at risk for HIV. Clinical trials in several countries are evaluating alternative approaches for mitigating the adherence challenges of daily oral dosing prevention regimens; these approaches include long-acting, injectable antiretroviral drugs such as cabotegravir and other long-acting agents as well as passive transfer of antibodies (*23*). 

The comprehensive portfolio of interventions in our HIV prevention toolbox served as the basis for a recent commentary in the lay press stating that there are “No more excuses. We have the tools to end the HIV/AIDS pandemic” (*24*). Despite these valuable advances in prevention of HIV infection, several challenges have been encountered in the optimal implementation of these modalities. These challenges include structural, legal, and social barriers resulting in inequalities of access to and uptake of HIV testing and treatment; lack of retention in care; social networks; stigma and discrimination; poor adherence to PrEP; limited access to special populations; and difficulty meeting the UNAIDS targets for enrolling persons living with HIV into treatment programs (*25**,**26*).

## Ending the HIV/AIDS Pandemic as an Epidemiologic and Global Public Health Phenomenon 

In contrast to addressing the needs of the individual person living with or at risk for HIV infection, achieving an end to the HIV/AIDS pandemic as an epidemiologic and global public health phenomenon requires a somewhat different approach. According to the latest UNAIDS statistics, in 2016, there were ≈1.8 million new HIV infections, 1.0 million AIDS deaths, and 36.7 million persons living with HIV (*27*). In 2014, with a goal of achieving an end to the pandemic, UNAIDS issued the 90-90-90 targets for HIV treatment scale-up by 2020. These targets mean that 90% of persons with HIV/AIDS have had their infection diagnosed, 90% of those with a diagnosis are receiving ART, and 90% of those receiving ART have achieved virus suppression (*28*). In real numbers, these targets translate to 33.0 million persons with HIV receiving a diagnosis, 29.7 million persons receiving ART, and 26.8 million persons receiving ART acheiving virus suppression (*26*).

UNAIDS-sponsored modeling exercises predict an end of the HIV/AIDS pandemic by 2030 if the 90-90-90 targets are achieved by 2020 and 95-95-95 targets are achieved with a decrease to 200,000 new infections among adults annually (*29**,**30*). Although substantial progress has been made, with certain countries meeting the 90-90-90 targets (*31*) and 53% of all persons living with HIV accessing ART in 2016, a critical global HIV treatment gap remains: of the 36.7 million HIV-infected persons, an estimated 17.2 million are not receiving ART. Of those receiving ART, virus is suppressed in only 44%. In addition, the Fast-Track Target, agreed on by the United Nations General Assembly of reducing the number of new HIV infections to <500,000 per year by 2020 and 200,000 per year by 2030 is not being met and is substantially off target. All subpopulations must be included in this targeting. Specifically, UNAIDS notes that from 2010 to 2016, the average annual decrease in HIV incidence worldwide was only 2.3% (*26*). UNAIDS recently issued its HIV Prevention 2020 Road Map, which outlines a 10-point action plan that focuses on the need for country-level actions to achieve a 75% reduction in new HIV infections and country-level achievement of that specific goal of the 90-90-90 targets (*25*).

## Summary 

From a theoretical standpoint, the goal of ending the HIV/AIDS pandemic is achievable; however, it will require additional and sustained resources to make available the already existing scientific advances on local, regional, national, and global levels. From a practical standpoint, accomplishing this goal will be a substantial challenge. In this regard, the essential components in the effort toward achieving this most challenging goal would probably be development of a moderately effective HIV vaccine together with optimal implementation of existing treatment and prevention modalities (*32*). As persons living with HIV or at risk of acquiring HIV who have access to treatment and prevention continue to benefit from the fruits of scientific advances, we must not take our sights off or waiver in our pursuit of the ultimate goal of ending the epidemic as a global health catastrophe.
